# Supplementary material for: Impact of Pre-Existing Immunity and Age on Antibody Responses to Live Attenuated Influenza Vaccine
Source: Vaccines (Basel). 2024 Aug 1;12(8):864. doi: 10.3390/vaccines12080864 (PMC11359048; doi:10.3390/vaccines12080864)
Supplement: Supplementary file 1 [file vaccines-12-00864-s001.zip › vaccines-3069874-supplementary.pdf]

Supplementary Table S1.A Dunn's test P values from the hemagglutination inhibition assay.

	Virus strains		
**Stratified Cohort**	**A/H1N1**	**A/H3N2**	**B/Mass**
Naïve Children	NS	NS	D0-D28 (0.0061)<br>D0-D56 (0.0011)
Primed Children <9	NS	NS	D0-D28 (0.0375)<br>D0-D56 (0.0297)
Primed Children >9	NS	NS	NS
Adults	NS	NS	D0-D28 (0.0052)<br>D14-D28 (0.0003)<br>D14-D56 (0.0435)

Supplementary Table S1.B Dunn's test P values from the microneutralization assay.

	Virus strains		
**Stratified Cohort**	**A/H1N1**	**A/H3N2**	**B/Mass**
Naïve	NS	NS	D0-D28 (0.0209)<br>D0-D56 (0.0115)
Primed Children <9	NS	D0-D56 (0.0127)	NS
Primed Children >9	D0-D28 (0.0311)	D0-D28 (0.0225)	NS
Adults	NS	D0-D14 (<0.0001)<br>D0-D28 (0.0001)<br>D0-D56 (<0.0001)	D0-D56 (0.0370)<br>D14-D56(0.0224)

Supplementary Table S1.C Dunn's test P values from the neuraminidase inhibition assay.

	Virus strains		
**Stratified Cohort**	**A/H1N1**	**A/H3N2**	**B/Yamagata**
Naive	NS	NS	NS
Primed Children <9	NS	NS	D0-D28 (0.005)<br>D0-D56 (0.0183)
Primed Children >9	NS	D14-D28 (0.0056)<br>D14-D56 (0.0225)	NS
Adults	D0-D14 (0.0265)<br>D14-D28 (0.0008)<br>D14-D56 (0.0001)	NS	D14-D56 (0.0035)

Supplementary Table S1.D Dunn's test P values from the hemagglutinin avidity assay.

	Virus strains		
**Stratified Cohort**	**A/H1**	**A/H3**	**B/H Mass**
Naïve	NS	NS	NS
Primed Children <9	D0-D28 (0.0031)<br>D0-D56 (0.0016)	NS	NS
Primed Children >9	NS	NS	NS
Adults	NS	NS	NS

Supplementary Table S1.E Dunn's test P values from full age group analysis according to assay.

	Virus strains		
**Assay**	**A/H1N1**	**A/H3N2**	**B/Mass**
Hemagglutination Inhibition	NS	D0-D28 (0.0079)<br>D0-D56 (0.0238)	D0-D28 (0.0001)<br>D0-D56 (0.0001)<br>D7-D28 (0.0001)<br>D7-D56 (0.0002)
Microneutralization	D0-D28 (0.0006)<br>D0-D56 (0.0246)	D0-D7 (<0.0001)<br>D0-D28 (<0.0001)<br>D0-D56 (<0.0001)	D0-D28 (0.0003)<br>D0-D56 (0.0001)<br>D7-D28 (0.0004)<br>D7-D56 (<0.0001)
Neuraminidase Inhibition	D0-D56 (0.0131)<br>D7-D28 (0.0047)<br>D7-D56 (<0.0001)	D7-D28 (0.0337)<br>D7-D56 (0.0337)	D0-28 (0.001)<br>D0-D56 (0.002)<br>D7-D28 (0.0036)<br>D7-D56 (0.0069)

Statistically significant P values ≤ 0.05 are specified. Day is denoted as D, and non-significant as NS.

Supplementary Table. S2. Geometric mean fold changes 56 days post LAIV vaccination compared to pre vaccination.

	A/H1N1					A/H3N2					B				
	HI	MN	NAI	HA Avi	NA Avi	HI	MN	NAI	HA Avi	NA Avi	HI	MN	NAI	HA Avi	NA Avi
**Naive Children < 9**	2.8	2.2	1.7	1.2	0.7	9.5	2.0	2.1	1.0	0.7	35.5	6.5	1.7	1.2	0.9
**Primed Children < 9**	1.5	2.1	1.5	1.9	0.9	1.5	3.2	1.4	1.1	0.6	2.5	2.1	1.9	0.9	1.0
**Primed Children > 9**	2.7	2.6	1.5	1.0	1.0	2.8	2.1	1.4	0.8	0.2	2.7	1.8	1.7	1.3	0.8
**Adults**	1.3	1.4	1.3	2.0	1.1	1.4	1.8	1.2	1.1	1.2	1.6	1.9	1.2	1.0	0.9
**All Age Groups**	1.7	1.8	1.5	1.6	0.9	2.0	2.1	1.4	1.0	0.9	3.1	2.3	1.6	1.0	0.9

Supplementary Figure S1. Full age group analysis of HI, MN, and NAI titre changes following LAIV vaccination.

Hemagglutination Inhibition (HI), Microneutralization (MN), and NAI (Neuraminidase Inhibiting) titre. Y axis are log 2 transformed, and values are expressed as geometric mean values with 95% confidence intervals.

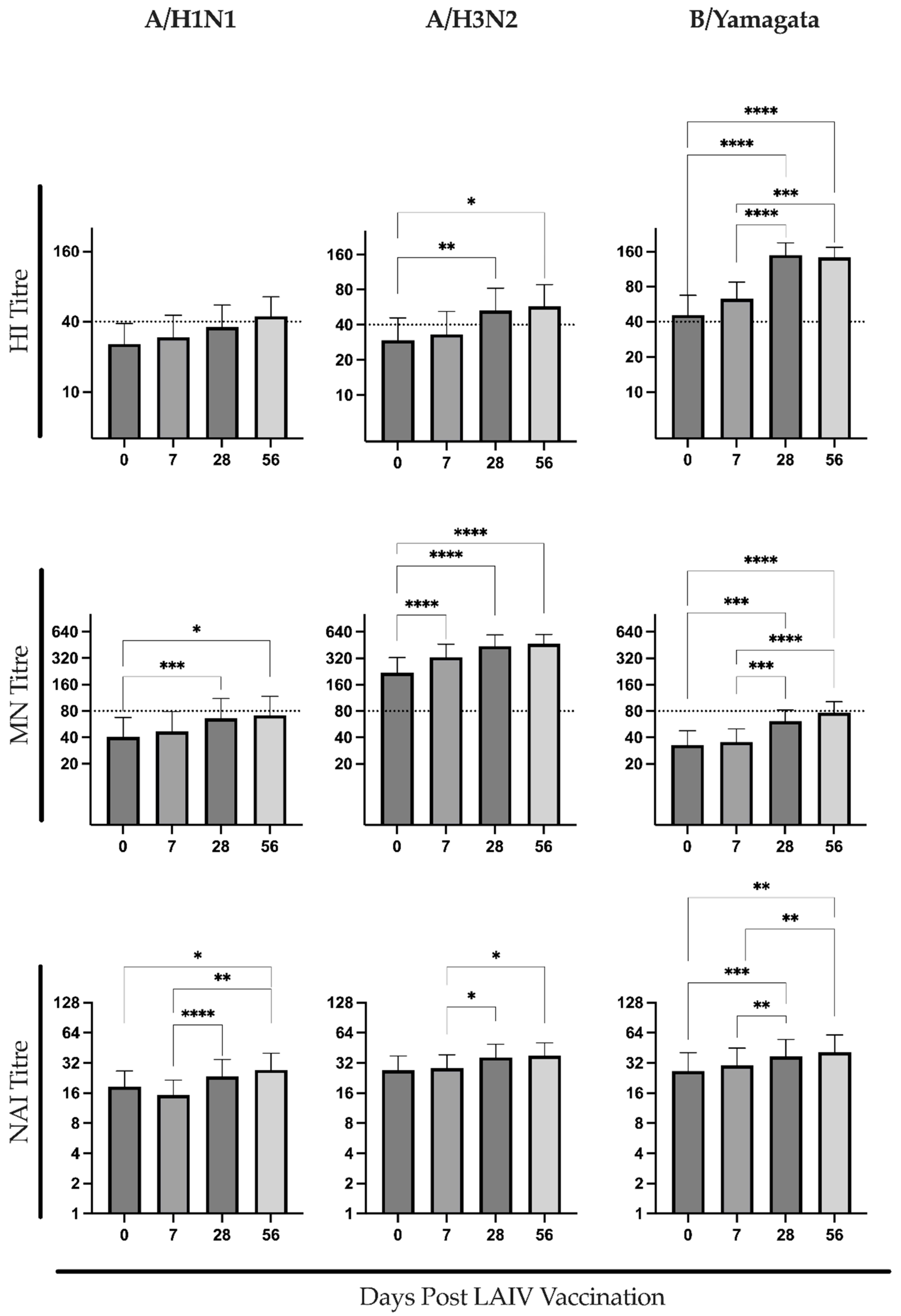

A/H1N1
A/H3N2
B/Yamagata
HI Titre
MN Titre
NAI Titre
Days Post LAIV Vaccination
